# Thoracic Endometriosis and Catamenial Pneumothorax: Imaging Pitfalls and an Integrated Diagnostic Approach

**DOI:** 10.3390/jcm15124517

**Published:** 2026-06-11

**Authors:** Marija Varnicic Lojanica, Stefan Ivanovic, Nikola Milic, Nikola Jovic, Nenad Rakic, Igor Pilic, Katarina Ivanovic, Maja Matijasevic, Dejana Rakic, Jovana Joksimovic Jovic, Milica Ivanovic

**Affiliations:** 1 Užice General Hospital, 31000 Užice, Serbia; maqy88@gmail.com (M.V.L.); maja.bunardzic@gmail.com (M.M.); 2 Obstetrics and Gynecology Clinic “Narodni Front”, 11000 Belgrade, Serbia; 3 Department of Gynecology and Obstetrics, Faculty of Medical Sciences, University of Kragujevac, 34000 Kragujevac, Serbia; docctorny@gmail.com (N.J.); dejavulovic@gmail.com (D.R.); 4 Clinical Center Kragujevac, Zmaj Jovina 30, 34000 Kragujevac, Serbia; 5 Senta General Hospital, 24400 Senta, Serbia; nenad.rakic@ymail.com; 6 Faculty of Medicine, University of Belgrade, 11000 Belgrade, Serbia; pilic.igor@yahoo.com; 7 Clinic for Gynecology and Obstetrics, University Clinical Center of Serbia, 11000 Belgrade, Serbia; ikatarina.1996@gmail.com; 8 Department of Physiology, Faculty of Medical Sciences, University of Kragujevac, 34000 Kragujevac, Serbia; jovana.joksimovic@fmn.kg.ac.rs; 9 Center of Excellence for Redox Balance Research in Cardiovascular and Metabolic Disorders, 34000 Kragujevac, Serbia

**Keywords:** thoracic endometriosis, catamenial pneumothorax, thoracic endometriosis syndrome, diagnostic imaging, magnetic resonance imaging, computed tomography, diagnostic pitfalls

## Abstract

Catamenial pneumothorax is a rare form of recurrent spontaneous pneumothorax occurring in women in temporal association with the menstrual cycle, most commonly within 72 h before or after the onset of menstruation, and is frequently encountered as part of thoracic endometriosis syndrome. Thoracic endometriosis represents an extrapelvic manifestation of endometriosis in which ectopic endometrial tissue may involve the pleura, diaphragm, lung parenchyma, or airways, leading to cyclic pleuropulmonary symptoms. The clinical spectrum includes catamenial pneumothorax, catamenial hemothorax, catamenial hemoptysis, and pulmonary endometriotic nodules. This narrative review critically analyzes the diagnostic challenges and limitations of imaging modalities in thoracic endometriosis, with particular emphasis on diagnostic delay, radiological pitfalls, and the discrepancy between morphological detection and etiological confirmation. Chest radiography and computed tomography are useful for documenting acute thoracic events, whereas magnetic resonance imaging may provide additional tissue characterization in selected cases, particularly when hemorrhagic or diaphragmatic lesions are suspected. However, imaging findings are often nonspecific, temporally variable, and insufficient to establish the diagnosis when interpreted in isolation. Recognition of thoracic endometriosis therefore requires correlation of imaging findings with menstrual cyclicity, gynecological history, clinical phenotype, and, when indicated, surgical and histopathological assessment. The available evidence remains limited by retrospective designs, small case series, inconsistent diagnostic criteria, and lack of validated thoracic-specific imaging pathways. Accordingly, an integrated clinical–radiological–surgical approach should be regarded as a pragmatic diagnostic framework rather than a validated algorithm. Such an approach may improve clinical suspicion, reduce diagnostic delay, and support more appropriate multidisciplinary management of this underrecognized condition.

## 1. Introduction

Catamenial pneumothorax (CP) represents a rare type of recurrent spontaneous pneumothorax that occurs in women in clear temporal association with the menstrual cycle, most commonly within 72 h before or after the onset of menstruation [1]. The term “catamenial”, derived from the Greek word “katamēnios” (καταμήνιος), meaning “monthly”, refers to clinical phenomena that recur cyclically in correlation with hormonal fluctuations. Although the temporal definition appears precise, the underlying pathophysiology and diagnostic implications of this entity are considerably more complex. CP most commonly occurs as part of thoracic endometriosis syndrome (TES), a rare but clinically significant condition characterized by the presence of functional endometrial tissue within thoracic structures, including the visceral and parietal pleura, lung parenchyma, diaphragm, and, less commonly, the airways. In this context, the term catamenial syndrome (CS) is used to describe the clinical manifestations temporally associated with the menstrual cycle, while TES denotes the underlying pathological entity. The term thoracic endometriosis primarily refers to the anatomical localization of the disease, whereas TES represents its clinical syndrome expressed through different phenotypic manifestations [2]. As in pelvic localization, ectopic endometrial tissue undergoes cyclic hormonally mediated changes, leading to recurrent pleuropulmonary manifestations that are most pronounced during the perimenstrual period [3]. The clinical spectrum of TES is highly heterogeneous and includes: CP (30–73%), catamenial hemothorax (CHt) (≈14%), catamenial hemoptysis (CHp) (≈7%), and pulmonary nodular lesions (≈6%) [3]. Although endometriosis as a systemic disease affects approximately 3–10% of women of reproductive age and 2–5% of postmenopausal women, thoracic involvement remains rare and is likely significantly underestimated in everyday clinical practice [2]. It is important to emphasize that the relative rarity of reported cases probably partly reflects diagnostic limitations rather than the true epidemiological frequency of the condition. Contemporary guidelines, including recommendations from the European Society of Human Reproduction and Embryology, increasingly recognize endometriosis as a systemic disease with the potential for extrapelvic manifestations, which requires a diagnostic approach that extends beyond traditional gynecologic frameworks and involves interdisciplinary evaluation of atypical presentations [3]. In this context, thoracic endometriosis represents a paradigmatic example of a condition in which failure to integrate gynecologic history and hormonal cyclicity into the evaluation of thoracic symptoms leads to substantial diagnostic delay. Despite the relatively low reported incidence, TES carries a disproportionately high diagnostic burden. In a considerable number of cases, a long interval exists between the onset of initial symptoms and the establishment of the final diagnosis, with recurrent thoracic events often interpreted as primary spontaneous pneumothorax or nonspecific pleuropulmonary pathology [4]. Such delays have direct clinical consequences, including repeated hospitalizations, repeated diagnostic procedures, and the absence of etiologically targeted therapeutic approaches.

A central problem in the existing literature is that imaging modalities often document the consequences of the disease rather than its underlying cause. Pneumothorax, hemothorax, pleural effusion, or transient parenchymal abnormalities can usually be detected, but their cyclic and hormonally mediated nature often remains unrecognized when imaging findings are interpreted without clinical context [5,6]. This distinction between morphological detection and etiological interpretation represents one of the main diagnostic challenges in thoracic endometriosis. Existing reports are frequently descriptive, with imaging findings presented without sufficient analysis of their clinical implications, temporal variability, or relationship to surgical confirmation [5,6]. This contributes to misattribution of findings and perpetuates diagnostic uncertainty.

Imaging modalities occupy an important position in the diagnostic evaluation of suspected thoracic endometriosis. However, their diagnostic value is limited by nonspecific and temporally variable findings [5,6]. No imaging modality, when considered in isolation, can reliably establish the etiological diagnosis of thoracic endometriosis [5,6]. Given the rarity of this condition, the heterogeneity of its clinical presentation, and the absence of universally accepted diagnostic criteria and standardized thoracic imaging algorithms, thoracic endometriosis remains an insufficiently defined entity in diagnostic radiology, contributing to persistent diagnostic uncertainty and variability in clinical management.

The aim of this narrative review is to critically analyze the diagnostic challenges and limitations of imaging methods in catamenial syndrome and thoracic endometriosis, with particular emphasis on mechanisms of diagnostic delay, radiological pitfalls, and the discrepancy between imaging-based detection and etiological confirmation. Specifically, this review aims to:

(1) summarize the imaging characteristics of thoracic endometriosis;
(2) highlight the limitations and interpretative pitfalls of chest radiography, computed tomography, and magnetic resonance imaging;
(3) propose a pragmatic diagnostic framework that integrates clinical history, menstrual cyclicity, imaging findings, and surgical assessment.

## 2. Materials and Methods

### 2.1. Study Design

This study was conceived as a narrative review of the literature aimed at analyzing the diagnostic challenges and limitations of imaging modalities in catamenial syndrome and thoracic endometriosis. A narrative approach was chosen due to the pronounced heterogeneity of the available literature, which is predominantly composed of case reports and case series, small retrospective cohort studies, expert reviews, and consensus-based recommendations, as well as the absence of homogeneous prospective studies that would allow a methodologically justified quantitative synthesis or meta-analysis. Therefore, this review was not designed as a formal systematic review, scoping review, or PRISMA-ScR-compliant evidence map. In such a context, an attempt at quantitative aggregation of data could create an appearance of methodological precision without corresponding clinical validity.

### 2.2. Literature Search Strategy

A structured literature search was conducted in the PubMed/MEDLINE, Scopus and Web of Science databases. The search was conducted to identify publications relevant to the diagnosis, imaging characteristics, and diagnostic pitfalls of catamenial syndrome and thoracic endometriosis. The search included studies published between January 2016 and January 2026, limited to articles written in English, available in full text, and conducted in human subjects. The search strategy was based on combinations of keywords and MeSH terms using Boolean operators (AND/OR). The following search string was used in PubMed/MEDLINE and adapted as appropriate to the syntax of Scopus and Web of Science: (“thoracic endometriosis” OR “thoracic endometriosis syndrome” OR “pulmonary endometriosis” OR “pleural endometriosis” OR “diaphragmatic endometriosis” OR “catamenial pneumothorax” OR “catamenial hemothorax” OR “catamenial hemoptysis”) AND (“diagnostic imaging” OR “computed tomography” OR “magnetic resonance imaging” OR “chest MRI” OR “radiology”). Additional studies were identified through manual screening of the reference lists of the included publications. Seminal older publications and relevant guideline or consensus documents were also considered when they provided essential historical, conceptual, diagnostic, or clinical context. The starting year of 2016 was chosen to capture the most recent decade of literature, reflecting contemporary imaging techniques, current diagnostic concepts, and modern multidisciplinary approaches to thoracic endometriosis.

### 2.3. Study Selection

The selection process was conducted in several predefined stages. Two authors independently screened the search results based on titles and abstracts, eliminating duplicates and studies that were clearly not relevant to the topic of the review. Disagreements regarding study inclusion were resolved through discussion and consensus. After the initial screening, studies that met the relevance criteria were analyzed in full text in order to assess their diagnostic, radiological, and clinical relevance to the aims of the review. Publications lacking a clear diagnostic or radiological context, studies with insufficiently described imaging findings, and studies focused exclusively on therapeutic outcomes without adequate diagnostic analysis were excluded from the final evaluation. Because this article was designed as a narrative review rather than a formal systematic or scoping review, study selection was guided by relevance to the predefined thematic focus of the manuscript rather than by formal PRISMA-ScR methodology.

### 2.4. Inclusion and Exclusion Criteria

Studies were included if they met at least one of the following criteria:

- Analyzed diagnostic criteria or radiological characteristics of thoracic endometriosis or catamenial syndrome;
- Described imaging patterns on computed tomography, magnetic resonance imaging, or chest radiography relevant to differential diagnosis;
- Identified diagnostic pitfalls, interpretative limitations, or factors contributing to delayed diagnosis;
- Examined the relationship between clinical presentation, menstrual cyclicity, imaging findings, and surgical or histopathological confirmation;
- Represented guideline, consensus, or expert-based documents with diagnostic, conceptual, or clinical implications for thoracic or extrapelvic endometriosis.

Studies were excluded if they focused exclusively on therapeutic outcomes without a diagnostic or imaging context, provided insufficient clinical or radiological detail, were not available in full text, were published in languages other than English, or involved non-human subjects.

### 2.5. Data Extraction and Narrative Synthesis

Data from the included studies were analyzed qualitatively. The following domains were extracted:

- type and localization of thoracic manifestation;
- radiological characteristics according to imaging modality;
- temporal association of symptoms or imaging findings with the menstrual cycle;
- relationship between imaging findings and surgical or histopathological confirmation;
- described diagnostic pitfalls and interpretative limitations;
- factors associated with delayed diagnosis;
- diagnostic or conceptual recommendations from guideline and consensus documents;
- reported evidence gaps and practical implications for clinical decision-making.

Data synthesis was conducted thematically and narratively, with the aim of identifying recurring patterns, typical and atypical radiological presentations, systemic diagnostic limitations, and clinically relevant points of uncertainty. Particular attention was given to the mismatch between radiological detection of thoracic events and etiological confirmation of thoracic endometriosis.

A quantitative meta-analysis was not performed due to marked heterogeneity in study design, diagnostic criteria, imaging protocols, outcome reporting, and the predominance of case reports, case series, and retrospective studies.

### 2.6. Methodological Limitations

The main limitation of this review lies in its narrative design. Although the literature search and study selection were structured, this article was not designed as a formal systematic review or scoping review and did not follow PRISMA-ScR methodology. Consequently, the findings should be interpreted within the methodological limits of a critical narrative synthesis. An additional limitation arises from the nature of the available evidence. The literature on thoracic endometriosis is dominated by case reports, small case series, retrospective studies, expert reviews, and consensus-based recommendations. This limits the generalizability of conclusions and increases the risk of publication bias, particularly because unusual, recurrent, severe, or surgically confirmed cases are more likely to be reported than mild or subclinical presentations. The heterogeneity of available studies also limits direct comparison across reports. Definitions of thoracic endometriosis syndrome, catamenial pneumothorax, and endometriosis-associated pneumothorax are not uniform. Imaging protocols, timing of imaging in relation to the menstrual cycle, use of MRI, surgical exploration, histopathological confirmation, and follow-up strategies vary substantially across studies. These factors prevent reliable standardization of diagnostic pathways.

Despite these limitations, a narrative approach was considered appropriate for integrating heterogeneous evidence and identifying clinically relevant diagnostic patterns, recurring pitfalls, and areas of uncertainty. The proposed diagnostic approach should therefore be interpreted as a flexible clinical decision-support framework rather than as a validated or strictly defined algorithm. Its purpose is to support suspicion and guide multidisciplinary evaluation in patients with suggestive clinical patterns, while recognizing that individualized interpretation remains necessary in each clinical case.

## 3. Clinical Spectrum of Catamenial Syndrome

The clinical manifestations of thoracic endometriosis may conceptually be grouped into pleural, parenchymal/endobronchial, and atypical phenotypes, reflecting differences in anatomical localization and underlying pathophysiological mechanisms. CS represents the clinical manifestation of TES and encompasses a heterogeneous spectrum of pleural and parenchymal lesions temporally associated with the menstrual cycle. Individual clinical entities such as CP, CHt, and CHp represent specific phenotypic manifestations within this syndrome [7,8]. In everyday clinical practice, this entity is often reduced to CP. Modern reviews clearly indicate that it represents a broader clinical continuum with multiple phenotypic patterns [7,9]. The phenotypic variability of CS reflects differences in the localization and depth of infiltration of endometrial implants, as well as in the degree of pleuropulmonary response [9,10]. This heterogeneity has direct diagnostic implications, particularly in the context of imaging modalities, whose sensitivity and specificity vary significantly depending on the dominant clinical phenotype. From an analytical perspective, manifestations of CS may be considered through a pleural phenotype (pneumothorax and hemothorax), a parenchymal/endobronchial phenotype, and atypical forms. Although this framework does not represent a formal classification, it allows a more precise understanding of diagnostic challenges and patterns of delayed diagnosis [7,8,11]. The clinical and radiological characteristics of the dominant phenotypes are presented in Table 1.

### 3.1. Pleural Phenotype: Catamenial Pneumothorax

CP represents the most common manifestation of CS and the dominant form of thoracic endometriosis in most published series [9]. It is defined as a spontaneous pneumothorax occurring in the perimenstrual period, most commonly within 72 h after the onset of menstruation, although the temporal pattern may be variable [9,12]. One of the most consistent clinical characteristics is the marked right-sided predominance, confirmed in multiple series and reviews [12,13]. This lateralization has been associated with anatomical and physiological factors, including diaphragmatic defects and the potential transdiaphragmatic migration of endometrial tissue [8,11]. Although it may represent a useful diagnostic clue, this lateralization is rarely interpreted in an etiological context in routine clinical practice. The clinical presentation of CP is almost identical to that of primary spontaneous pneumothorax, with acute dyspnea and pleuritic chest pain being the dominant symptoms [9,13]. This overlap in symptomatology contributes significantly to diagnostic delay. In the absence of targeted history-taking and systematic evaluation of symptom cyclicity, individual episodes are often treated as isolated events without etiological correlation [9,14]. In practice, suspicion of CS is most often raised retrospectively, after recurrent episodes reveal a pattern of pneumothorax occurring during the same phase of the menstrual cycle [9,14]. This model of “retrospective recognition” highlights the gap between acute thoracic management and the integration of gynecological information in the diagnostic process. It is important to note that available studies do not consistently report temporal patterns and incidence of CP, which further complicates the standardization of diagnostic criteria and contributes to variability in clinical management.

### 3.2. Pleural Phenotype: Catamenial Hemothorax

CHt represents a less common but potentially more severe manifestation of CS [7,9]. It is characterized by the accumulation of hemorrhagic content in the pleural space, most often unilateral and predominantly right-sided [8,9,11]. The clinical presentation includes chest pain, progressive dyspnea, and, in more severe cases, signs of acute blood loss [7]. Despite the severity of the clinical presentation, etiological identification is frequently lacking. In the absence of trauma, coagulopathy, or malignancy, hemothorax is often classified as idiopathic without consideration of its cyclic association with the menstrual cycle [8,9,11]. This paradox, in which greater clinical severity does not necessarily lead to faster etiological clarification, represents an important diagnostic pitfall within CS. As with CP, only the recurrence of episodes and retrospective analysis of symptom cyclicity typically lead to suspicion of thoracic endometriosis [9,14].

### 3.3. Parenchymal and Endobronchial Phenotype: Catamenial Hemoptysis

CHp represents the rarest but conceptually the most distinct form of CS, as it directly reflects cyclic hemorrhage within the pulmonary parenchyma or airways [8,10,11]. Clinically, it manifests as recurrent episodes of hemoptysis temporally synchronized with the menstrual cycle, most commonly during the first days of menstrual bleeding [10]. Unlike pleural phenotypes, in which symptomatology overlaps with considerably more common thoracic conditions, CHp has greater diagnostic specificity precisely because of its temporal regularity. However, in real clinical practice, hemoptysis initially triggers a broad differential diagnostic work-up that includes infectious, vascular, and neoplastic causes, while thoracic endometriosis is rarely suspected at the initial stage [8,11]. The literature indicates that the diagnosis of pulmonary endometriosis in this context is most often established only after extensive evaluation and systematic exclusion of other causes of hemoptysis [8,10,11]. This pattern suggests that even in phenotypes with a clear cyclic component, etiological clarification requires a high level of clinical suspicion and the integration of gynecological history with pulmonary evaluation. From a pathophysiological perspective, the presence of endometrial tissue within the pulmonary parenchyma or endobronchially leads to cyclic microhemorrhage, which may result in transient radiological changes that are often subtle or absent outside the perimenstrual period. This dynamic nature of the lesions further complicates radiological correlation and contributes to variability of imaging findings between episodes.

### 3.4. Atypical and Subclinical Presentations: Limits of Recognition

Beyond the classic pleural and parenchymal phenotypes, CS may present with forms that do not fulfill standard clinical patterns and remain below the threshold of diagnostic suspicion [7,9]. These forms include cyclic thoracic or scapular pain without objective pneumothorax, minimal or transient pleural effusions, as well as episodes of nonspecific dyspnea that resolve spontaneously. In such situations, the absence of a clear radiological finding significantly reduces the likelihood that TES will be considered as a possible etiology. As a result, diagnosis is often delayed and symptoms are attributed to musculoskeletal, functional, or nonspecific thoracic disorders [14]. This part of the spectrum is probably the most underestimated in the literature, as the majority of published reports describe clinically overt or complicated forms of the disease [7,9]. Subclinical and oligosymptomatic forms therefore remain underrepresented in publications, which contributes to underestimation of the true frequency of thoracic involvement in patients with endometriosis. From a diagnostic perspective, these forms most clearly demonstrate the limitations of relying exclusively on imaging modalities. In the absence of pronounced morphological changes, clinical assessment and careful history-taking remain the key elements of the diagnostic process.

### 3.5. Temporal Association of Symptoms with the Menstrual Cycle as a Central Diagnostic Principle

The temporal association of thoracic symptoms with the menstrual cycle represents the fundamental diagnostic criterion of CS and the most reliable clinical indicator of suspected thoracic endometriosis [9,12]. Available data indicate that this pattern is not entirely uniform. The interval between the onset of menstruation and the appearance of symptoms may vary, and in some patients the temporal association is not strictly confined to the classic perimenstrual window [9]. This variability contributes to misinterpretation in clinical practice, particularly when the history is obtained unsystematically or without explicit consideration of symptom cyclicity [12]. The clinical significance of temporal association lies not in a rigid temporal definition but in the recognition of a recurring cyclic pattern consistently linked to the menstrual cycle. In practice, a structured clinical history focusing on the relationship between symptoms and phases of the menstrual cycle often precedes radiological confirmation and represents a crucial step in establishing the underlying etiology and final diagnosis. This principle is particularly important in atypical and subclinical forms of the disease, where imaging findings may be minimal, transient, or entirely absent [9,12].

## 4. Imaging Characteristics and Diagnostic Limitations in Thoracic Endometriosis

The application of imaging modalities in TES occupies a central position in the diagnostic process. Their ability to determine etiology remains limited. Unlike most pleuropulmonary entities, in which the morphological pattern often directly suggests the underlying etiology, imaging findings in TES most commonly reflect secondary consequences of cyclic hemorrhage and pleural reaction, while endometriotic implants are difficult to visualize or remain undetectable [15,16]. This morphological–etiological discrepancy represents the central diagnostic problem in TES. The key issue is not only the nonspecific nature of imaging findings, but also their interpretation without adequate clinical context—imaging findings are usually classified within more common entities, which is the leading cause of diagnostic error. Pneumothorax or hemothorax can be readily identified, yet their cyclic nature remains unrecognized when imaging findings are interpreted without clinical context [17,18]. Therefore, imaging primarily documents the clinical event without elucidating the etiology of detected lesions. Modern literature reviews indicate that the diagnosis of TES can only be established through the integration of three components: the clinical phenotype, the temporal association of symptoms with the menstrual cycle, and interpretation of imaging findings with full awareness of their limited specificity [16,18].

### 4.1. Chest Radiography: Initial Assessment Without Etiological Differentiation

CXR represents the first diagnostic step in the evaluation of acute thoracic symptoms. Its role in TES is clearly defined: detection of pneumothorax, assessment of the extent of pleural effusion, and identification of hydropneumothorax [17,19]. However, radiographic findings are insufficient for determining the etiology of TES. Pneumothorax caused by endometriosis does not exhibit specific radiographic characteristics that would distinguish it from primary spontaneous pneumothorax or secondary pneumothorax of other etiologies. The same applies to hemothorax, where CXR demonstrates the presence of pleural fluid. In the parenchymal phenotype, particularly in CHp, CXR may remain normal even during symptomatic episodes, as microhemorrhages or small subsegmental lesions remain below the spatial resolution of the modality [15]. Between episodes, radiographic findings are often completely normal [16]. Therefore, CXR has high value in the acute assessment of patient stability. A normal or nonspecific radiographic finding does not reduce the clinical probability of TES in patients with recurrent, cyclic thoracic symptoms. A comparative overview of the diagnostic capabilities of individual imaging modalities is presented in Table 2.

### 4.2. Computed Tomography: High Sensitivity for the Event and Limited Ability to Identify the Substrate

CT represents the imaging modality of choice in the evaluation of pneumothorax and hemothorax. It allows precise quantification of pleural gas, assessment of the density of pleural content, and identification of associated parenchymal changes, including subpleural consolidations and cystic lesions [17,19]. Reported CT findings in TES include ground-glass opacities, subtle subpleural infiltrates, bullous changes, and focal pleural thickening [15,16,23]. These CT findings reflect alveolar microhemorrhage, transient inflammatory reaction, or secondary structural alterations resulting from recurrent pulmonary atelectasis. None of these findings is specific for endometriosis, and they substantially overlap with infectious processes, vascular conditions, or autoimmune diseases. In cases of diaphragmatic involvement, CT may demonstrate subtle defects or nodular changes, although in small implants or stromal lesions without pronounced hemorrhagic content the sensitivity of the modality remains limited [18,20]. In a considerable number of cases, CT findings remain normal despite subsequent thoracoscopic confirmation of disease. A particular diagnostic challenge arises from the cyclic dynamics of imaging findings. Hemorrhagic or inflammatory changes evident during menstruation may partially or completely regress during subsequent phases of the menstrual cycle, resulting in false-negative follow-up CT findings [16]. This phenomenon highlights the importance of temporal alignment between imaging acquisition and clinical context. CT reliably identifies the type and extent of pathological changes but, in most cases, is not able to determine their etiology. Diagnostic characteristics of imaging modalities and surgical exploration are summarized in Table 3.

### 4.3. Magnetic Resonance Imaging: Tissue Characterization, Timing, and the Real Capabilities of the Method

MRI is the most specific non-invasive imaging modality for tissue characterization of pathological changes in thoracic endometriosis, primarily because of its ability to detect hemorrhagic content and its superior contrast resolution in the visualization of soft-tissue abnormalities [15,21,22]. Unlike CT, which predominantly detects structural events such as the presence of gas, fluid, or atelectasis, MRI can more precisely characterize intralesional content and localization of abnormalities (diaphragmatic, pleural or parenchymal) [16,18]. The diagnostic value of MRI in TES depends largely on an appropriately designed imaging protocol and proper temporal alignment with the symptomatic phase of disease. The minimum recommended set of sequences includes T1-weighted images with and without fat suppression, high-resolution T2-weighted sequences, and, when needed, DWI. The most typical MRI finding in thoracic endometriosis is hyperintensity of lesions on T1-weighted sequences, particularly on fat-suppressed images, while signal intensity on T2-weighted sequences is variable and depends on the phase of hemorrhage, the degree of fibrosis, and the local inflammatory reaction. The absence of marked contrast enhancement is not unusual since lesions often represent chronic hemorrhagic-fibrotic implants without active neovascularisation [21,22]. In diaphragmatic disease, MRI may demonstrate nodular or subtle linear changes, focal defects, or areas of T1 hyperintensity corresponding to hemorrhagic implants [18,20]. Imaging during menstruation or immediately before it may increase conspicuity of hemorrhagic components, whereas in the intermenstrual phase partial resorption may reduce lesion visibility and contribute to false-negative studies [16,18]. Despite its superior tissue characterization, MRI is not uniformly sensitive across all phenotypes of thoracic endometriosis, particularly superficial pleural lesions, thin diaphragmatic fenestrations, and stromal implants without pronounced hemorrhagic content [20,21]. In a substantial number of cases, definitive confirmation of thoracic endometriosis still relies on thoracoscopy [16,18,20,21,22].

### 4.4. Video-Assisted Thoracic Surgery

Video-assisted thoracic surgery (VATS) represents the gold standard for both diagnosis and treatment of CP, enabling a range of interventions depending on lesion characteristics [2,20]. Direct visualization of pleural implants, diaphragmatic defects, or fenestrations, together with histopathological confirmation, enables definitive diagnosis of thoracic endometriosis [18,20]. Superficial endometriotic implants may be treated with ablative techniques, whereas deeper lesions typically require surgical excision, including wedge resection or, in selected cases, more extensive procedures. Adjunctive pleurodesis, either chemical or mechanical, has been associated with a reduction in recurrence rates following VATS. Furthermore, histological confirmation of endometrial tissue, combined with postoperative hormonal therapy, may contribute to improved long-term disease control and reduction in recurrence [2,7,20].

### 4.5. Diaphragmatic Endometriosis: An Anatomical and Radiological Challenge

Diaphragmatic involvement represents a central pathophysiological component of TES and a potential source of recurrent pneumothorax, hemothorax, and pleural reactions [18,20]. Despite this, the diaphragm remains one of the least thoroughly evaluated anatomical structures in routine thoracic imaging. Nevertheless, several studies emphasize systematic underestimation of disease extent when imaging findings are compared with intraoperative observations [18,20]. Diaphragmatic fenestrations may be microscopic or functional without clear visualization on imaging. This discrepancy between radiological and surgical findings represents one of the key diagnostic pitfalls and explains why multimodal imaging does not guarantee etiological confirmation [18].

### 4.6. Synthetic Overview of Chapter 4

Overall, imaging in thoracic endometriosis is essential for detecting complications and supporting clinical decision-making, but its etiological specificity remains limited. These limitations are particularly relevant in the differential diagnosis of spontaneous pneumothorax in women of reproductive age, summarized in Table 4. In clinical practice, imaging findings alone rarely establish a definitive diagnosis, but rather serve as an initial indicator that, in the presence of an appropriate clinical context, guides further diagnostic evaluation.

## 5. Pragmatic Diagnostic Pathway and Systemic Diagnostic Pitfalls

Despite the growing number of publications and advances in imaging techniques, TES and CS continue to be characterized by prolonged diagnostic intervals and frequent initial misinterpretation of imaging findings [16,18,24]. This reflects a mismatch between the temporal and clinical dynamics of the disease and the organization of the diagnostic process. Patients most commonly first present to pulmonologists, thoracic surgeons, or emergency departments with acute pneumothorax or hemothorax, while gynecological history is not systematically incorporated into the initial evaluation [17,24]. As a result, the diagnostic process remains fragmented, and etiological recognition of the disease is delayed.

### 5.1. First Diagnostic Node: When to Suspect Thoracic Endometriosis Syndrome

The key moment in the diagnostic process is the recognition of situations that require expanded evaluation. Based on available case series and systematic reviews, the following elements significantly increase the probability of thoracic endometriosis [16,18,24,25]:

- spontaneous pneumothorax in a woman of reproductive age without clear underlying pulmonary disease.
- recurrent episodes of right-sided pneumothorax.
- hemothorax without trauma or coagulopathy.
- cyclic hemoptysis.
- temporal association of symptoms with the menstrual cycle.

None of these factors individually is diagnostically determinant. Their combination, particularly in the context of recurrence, should prompt targeted history-taking and structured clinical evaluation [16,18]. At this stage, imaging has a confirmatory rather than etiological role. CXR and CT detect pathological changes but do not clarify their etiology [17,19]. The proposed diagnostic approach aims to integrate the temporal pattern of symptoms with imaging findings and enable their interpretation within the appropriate clinical context.

### 5.2. Second Diagnostic Node: Document the Event or Search for the Substrate?

After the initial detection of pneumothorax or hemothorax, the clinical work-up diverges into two parallel pathways:

1. Acute management and assessment of patient stability.
2. Etiological stratification.

In clinical practice, the second pathway is often delayed or completely omitted. Chest CT reliably demonstrates the extent of pleural gas or fluid, subpleural changes, and potential parenchymal abnormalities but lacks sufficient specificity in the context of TES [16,17,23]. It is precisely at this stage that the most common diagnostic error occurs: imaging findings are interpreted as sufficient for establishing a diagnosis without consideration of the broader clinical context. Contemporary studies also emphasize that a negative or nonspecific CT finding does not exclude TES and that the diagnostic process should not end at the level of detecting complications [16,18].

### 5.3. Third Diagnostic Node: Selective Use of MRI and Evaluation of the Diaphragm

In cases of recurrent episodes or persistent clinical suspicion, MRI becomes the next rational diagnostic step, particularly when diaphragmatic involvement or a hemorrhagic component is suspected [21,22]. However, its diagnostic value remains context-dependent. Superficial lesions and microscopic diaphragmatic fenestrations may remain undetected even with appropriately performed imaging [20]. It is important to emphasize that excessive reliance on “ideally timed” imaging during menstruation does not guarantee diagnostic confirmation, since the dynamics of lesions may vary considerably between individuals [16,18].

### 5.4. Fourth Diagnostic Node: Thoracoscopy as the Reference Standard for Diagnostic Confirmation

In situations with recurrent episodes or a high clinical index of suspicion, thoracoscopy remains the diagnostic and therapeutic standard for confirming the diagnosis of TES [18,20,25]. Comparisons between radiological and intraoperative findings in multiple case series have consistently demonstrated systematic underestimation of disease extent when assessed by imaging modalities [18,20]. This discrepancy does not represent a failure of imaging but rather reflects the limitations of noninvasive diagnostic methods in detecting microscopic and functional lesions.

### 5.5. Systemic Diagnostic Pitfalls

Based on the available analyses and clinical series, diagnostic errors in TES most commonly arise through three principal mechanisms [16,24,26]:

1. fragmentation of medical history—symptom cyclicity is not assessed systematically;
2. morphological reductionism—imaging findings are interpreted without etiological context;
3. false reassurance from negative imaging—normal CT or MRI findings lead to premature exclusion of the disease.

Recent reviews of technical and interpretative pitfalls further emphasize that insufficient attention directed toward the right hemidiaphragm, subpleural regions, and transient changes may contribute to false-negative imaging findings [26]. Taken together, these mechanisms help explain why the diagnostic process in TES often remains fragmented and why an integrated approach is required, as discussed in the following section.

### 5.6. Overlap with Other Pleuropulmonary Entities: Differential Diagnostic Complexity

One of the central reasons for diagnostic uncertainty in TES lies in the frequent overlap of its clinical and radiological features with considerably more common pleuropulmonary disorders. Spontaneous pneumothorax in women of reproductive age is most often initially interpreted in the context of primary spontaneous pneumothorax, bullous lung disease, lymphangioleiomyomatosis, or rare genetic syndromes, whereas hemothorax without trauma is usually considered within the spectrum of vascular, coagulation-related, or neoplastic causes [16,18,25]. The problem lies in the absence of consideration of a catamenial pattern. When temporal association with the menstrual cycle is not systematically evaluated, imaging findings—regardless of their precision—tend to be interpreted within the framework of more common etiological categories [16,26]. In the parenchymal phenotype, transient ground-glass opacities or subpleural infiltrates are easily attributed to infection, microembolization, or inflammatory processes [23,26]. Contemporary reviews of technical and interpretative pitfalls emphasize that identical radiological patterns may represent different pathophysiological processes and that clinical context is decisive in etiological attribution [26].

### 5.7. Structural Causes of Diagnostic Delay

Prolonged time to diagnosis represents a consistent finding in large case series and review studies [16,18,24]. This phenomenon cannot be explained solely by the rarity of the disease. The key factor lies in fragmentation of the diagnostic process. Patients are initially managed in thoracic or emergency care settings where the priority is stabilization of the acute event. After treatment of pneumothorax or hemothorax, the process often ends without further etiological evaluation, particularly when imaging findings are nonspecific or negative [17,24]. An additional factor is reliance on negative imaging findings as evidence for absence of disease. As previously discussed, CT and MRI have limited sensitivity for microscopic or superficial lesions, especially diaphragmatic ones [18,20,21]. In this context, a negative imaging result may create false diagnostic reassurance and prolong etiological clarification. Long-term follow-up series clearly show that diagnosis is often established only after multiple recurrences and thoracoscopic exploration, when implants or fenestrations previously undetected by imaging are finally identified [18,20,24].

### 5.8. The Gap Between Imaging, Intraoperative Findings, and Histopathology

Thoracoscopy is often considered the “gold standard” in the diagnosis of TES, but it is not absolutely reliable in all cases. Histopathological confirmation may be absent due to the microscopic nature of lesions, predominant fibrosis, or the lack of a typical glandular component [25,27]. Radiological–pathological correlations indicate that imaging systematically underestimates disease extent, particularly in superficial pleural implants and diaphragmatic fenestrations. At the same time, intraoperative findings may reveal nonspecific changes without clear histological confirmation of endometrial tissue. This discrepancy indicates that the diagnosis of TES requires correlation of findings across different diagnostic methods rather than reliance on a single modality [18,20,25].

### 5.9. Synthetic Conclusion of Section 5

In summary, the diagnostic difficulty of thoracic endometriosis reflects the combination of nonspecific imaging findings, temporal variability of disease expression, and fragmentation of the diagnostic pathway. Accordingly, imaging should be understood as an essential component of a diagnostic pathway, rather than as an independent means of etiological confirmation.

## 6. Integrated Diagnostic Approach in Suspected Thoracic Endometriosis

TES should be approached not as an isolated radiological, gynecological, or surgical diagnosis, but as a structured process including clinical phenotype, temporal symptom pattern, imaging findings, and, when required, surgical verification. Available evidence indicates that no single modality—including advanced imaging techniques or thoracoscopic exploration—can reliably establish the etiology on its own, making an integrated approach essential [20,25,28,29]. Contemporary reviews further show that fragmentation of clinical assessment, radiological interpretation, and surgical decision-making contributes substantially to diagnostic delay and recurrence in TES [28,29].

### 6.1. Clinical “Red Flags” and Situations That Should Raise Suspicion of Thoracic Endometriosis

Establishing clinical suspicion represents the foundation of the diagnostic pathway. TES remains systematically underestimated precisely because its manifestations—pneumothorax, hemothorax, hydropneumothorax, or hemoptysis—are initially interpreted within the framework of more common pleuropulmonary entities without consideration of the specific pattern of occurrence [25,30,31]. The most relevant clinical “red flags” include:

- recurrent spontaneous pneumothorax in women of reproductive age.
- marked right-sided predominance.
- recurrence of events in the perimenstrual period.
- association with pelvic symptomatology.

Evidence clearly indicates that CP represents an entity with specific epidemiological and anatomo-pathophysiological characteristics distinct from non-catamenial spontaneous pneumothorax [32]. The absence of strictly defined cyclicity does not exclude TES. The cyclic pattern may be recognized retrospectively, partially expressed, or masked by hormonal therapy [28,33]. Therefore, diagnostic suspicion must arise from a composite clinical pattern rather than from reliance on a single clinical event.

### 6.2. Targeted History and Gynecological Context as a Diagnostic Multiplier

Following initial suspicion, targeted history-taking represents a key element in the diagnostic pathway. TES rarely occurs in isolation, and in a significant proportion of cases it is associated with pelvic endometriosis, often of higher stage [28,32]. Information regarding dysmenorrhea, chronic pelvic pain, infertility, or previously confirmed endometriosis has substantial diagnostic value even when thoracic imaging findings remain nonspecific [28]. At the same time, the literature demonstrates that pelvic endometriosis is not infrequently diagnosed only after thoracic manifestations appear [32]. Additional diagnostic value may be provided by evaluation of response to hormonal suppression. Resolution of cyclic thoracic symptoms following initiation of therapy may represent a confirmation of the hormone-dependent nature of the process even in the absence of histological verification [33,34]. This principle further supports the concept that the diagnosis of TES is not exclusively morphological but rather a clinical–biological category.

### 6.3. The Role of Imaging in the Diagnostic Pathway: Confirmation of the Event, Not the Etiology

Imaging plays a central but conceptually limited role in the diagnostic pathway of TES. Systematic reviews show that CT and MRI reliably demonstrate morphological changes—pneumothorax, pleural effusion, hemothorax, or parenchymal infiltrates—whereas the endometriotic implant itself most often remains undetected [25,28,35]. The same imaging pattern may correspond to infection, thromboembolism, or malignancy, which further complicates the interpretation of imaging findings, while a negative finding does not exclude the disease [26,27]. This problem is particularly pronounced in diaphragmatic lesions and microscopic fenestrations, which are frequently identified intraoperatively but remain undetected by imaging modalities in a substantial number of cases [20,21,28]. Consequently, the role of imaging within an integrated diagnostic approach is threefold:

1. documentation of the acute event.
2. exclusion of alternative diagnoses.
3. support of clinical suspicion in the appropriate context.

Thus, imaging should not be regarded as an independent means of etiological confirmation, but rather as a key component of a diagnostic pathway [28,35].

## 7. Therapeutic Implications of Accurate and Timely Diagnosis

Although the central focus of this review is the diagnostic framework of TES, its therapeutic implications represent a crucial determinant of clinical outcome. Available data consistently show that diagnostic accuracy directly determines the extent of surgical intervention, the indication for hormonal suppression, and the risk of long-term recurrence. Conversely, delayed or partial diagnosis results in fragmented management, repeated interventions, and progressive chronification of the clinical course [28,29,36,37,38]. Therapeutic strategy in TES must therefore be phenotype-driven, sequential, and based on assessment of recurrence risk.

### 7.1. Breaking the Cycle of Repeated Interventions

In women of reproductive age with recurrent pneumothorax or hemothorax, repetition of standard thoracic procedures without etiological clarification leads to cumulative morbidity without effective disease control. Contemporary reviews of extragenital endometriosis emphasize that such a fragmented approach represents a major cause of prolonged diagnostic and therapeutic delay [37,39]. Timely diagnosis fundamentally changes the therapeutic paradigm: instead of episodic management of complications, an etiologically directed strategy is implemented with the aim of reducing future events.

### 7.2. Surgical Strategy: Who, When, and to What Extent

Available evidence indicates that therapeutic decisions should be individualized according to the clinical scenario. In patients presenting with a first episode of pneumothorax without clear clinical “red flags” suggestive of thoracic endometriosis, standard thoracic management with careful follow-up may be appropriate. In such cases, particular attention should be paid to the possible temporal association of symptoms with the menstrual cycle, as recognition of cyclicity may provide an important diagnostic clue.

In contrast, in patients with recurrent pneumothorax or in situations where clinical suspicion of TES is high, surgical exploration using VATS is generally recommended. During such procedures, systematic inspection of the pleura together with careful evaluation of the diaphragm is considered essential in order to identify potential diaphragmatic defects or pleural implants that may otherwise remain undetected. Meta-analytical data and retrospective cohort studies indicate that surgical interventions incorporating diaphragmatic resection or reconstruction, combined with an appropriate pleural strategy, are associated with significantly lower recurrence rates compared with limited procedures directed solely at the pulmonary parenchyma [29,36]. These observations support the concept that CP should not be interpreted as a primary pulmonary disorder, but rather as a diaphragmatic–pleural manifestation of endometriosis with specific operative implications [28]. It should be emphasized, however, that the available evidence is largely derived from retrospective analyses and meta-analyses rather than randomized controlled trials. Despite this limitation, the consistency of findings across multiple cohorts provides meaningful clinical guidance for surgical decision-making [29,36].

### 7.3. Hormonal Suppression: Selective but Essential

The role of hormonal suppression must be precisely defined:

- As an adjuvant following adequate surgical therapy, it significantly reduces the risk of recurrence [29,38,40].
- As monotherapy, it may be considered in mild phenotypes or when surgery is not indicated, but its long-term effectiveness is limited in patients with significant diaphragmatic lesions [28,29].

Contemporary data on recurrence-free survival confirm the superiority of a combined approach compared with surgery alone [34]. This effect is not only statistically significant but also clinically relevant in long-term follow-up. Hormonal suppression may also provide additional value as a functional indicator of the hormone-dependent nature of the process, but it should not replace anatomical correction of the pathological substrate in high-risk patients.

### 7.4. Prevention of Recurrence and Modification of the Natural Course of Disease

The most important therapeutic implication of an accurate diagnosis is reduction in recurrence. An integrated model—systematic surgery with diaphragmatic evaluation combined with postoperative hormonal suppression—shows the lowest recurrence rates of pneumothorax reported in the available literature [29,36,38,40]. In contrast, partial approaches lead to repeated hospitalizations and progressive chronification of symptoms. The long-term clinical burden includes not only medical but also psychosocial consequences, which are particularly pronounced in extragenital forms of the disease [37,38]. Early recognition of TES therefore alters the natural course of the disease—from a recurrent and fragmented condition toward a controlled and structured clinical trajectory.

### 7.5. Multidisciplinary Standard of Care

Optimal management of TES requires coordinated collaboration between thoracic surgery, gynecology, and radiology. A multidisciplinary model enables:

- appropriate planning of the operative strategy.
- rational use of hormonal therapy.
- structured long-term follow-up.

Within this framework, therapeutic decision-making does not represent an isolated action but rather a continuation of the integrated diagnostic process [28,37,38].

In real-world clinical settings, the application of such an approach is often limited by the availability of diagnostic modalities and organizational constraints. In particular, optimal timing of MRI in relation to the menstrual cycle is not always feasible, which may reduce its diagnostic value. In practice, the diagnostic process most often relies on a combination of available imaging findings, clinical suspicion, and patient history, with a stepwise progression toward more invasive methods when diagnostic uncertainty persists. Formal cost-effectiveness data for imaging-based pathways in TES are lacking, and real-world implementation is influenced primarily by local availability of imaging modalities, timing constraints, and multidisciplinary coordination.

### 7.6. Synthetic Conclusion of Chapter 7

Accurate and timely diagnosis of thoracic endometriosis has immediate and measurable therapeutic implications. It enables a transition from episodic management of complications toward a pathophysiologically oriented, phenotype-driven therapeutic model. Available evidence, although predominantly retrospective, consistently indicates that the combination of systematic surgical intervention with mandatory diaphragmatic evaluation and postoperative hormonal suppression provides the most stable long-term disease control [29,34,36,38,40]. In this context, integrated diagnosis represents not merely an academic ideal but a prerequisite for rational therapy and recurrence prevention in this complex and frequently underrecognized condition. Therapeutic decision-making should be individualized and phenotype-driven. A structured overview of phenotype-oriented therapeutic strategies is summarized in Table 5.

## 8. Limitations of the Available Evidence and Future Research Directions

Despite the growing number of publications on TES, this entity still lacks uniformly defined diagnostic and therapeutic protocols. The available evidence remains predominantly retrospective, phenotypically heterogeneous, and methodologically inconsistent, which limits its external validity and applicability in clinical practice [38,39,40,41,42,43,44,45,46]. These limitations represent a central barrier to the development of standardized diagnostic and therapeutic recommendations. Foundational studies established the principal clinical phenotypes and diagnostic associations of TES, whereas more recent evidence has increasingly highlighted the variability of imaging presentations, the limits of standard diagnostic pathways, and the need for integrated multidisciplinary interpretation [38,43,44].

### 8.1. Phenotypic Heterogeneity as a Central Methodological Weakness

One of the key limitations of existing studies is the absence of clear phenotypic stratification. CP, CHt, and CHp are often analyzed together despite differences in pathophysiology, clinical course, and therapeutic implications [43,44]. Such an approach leads to results that do not allow precise comparison of outcomes nor the development of phenotype-specific algorithms. In many studies, the same terminological framework includes isolated diaphragmatic fenestrations, combined pleuro-diaphragmatic lesions, and parenchymal manifestations without adequate differentiation in outcome analyses [39,44]. Without clear phenotype classification, therapeutic recommendations remain partially generalized and center-specific.

### 8.2. Surgical Variability and the Absence of Standardized Protocols

Data on the surgical management of TES are based almost exclusively on retrospective series and cohorts with considerable variability in operative techniques. The question of routine diaphragmatic repair remains a subject of debate [39]. While some authors advocate systematic reconstruction or covering techniques [45], others prefer an individualized approach depending on intraoperative findings. The absence of a standardized surgical algorithm complicates interpretation of recurrence rates and the identification of independent predictors of outcome. Randomized controlled trials are virtually nonexistent, and multicenter prospective studies are rare, which further limits the level of evidence.

### 8.3. Limitations of Imaging Evidence and the Need for Standardization

Data from the literature on the use of imaging methods in TES are predominantly focused on the detection of complications of this condition, while etiological characterization of lesions remains secondary [39,44]. Although MRI shows greater specificity in detecting hemorrhagic and diaphragmatic lesions, its diagnostic value depends on protocol design, cycle timing, and the experience of the radiologist [41,46]. In contrast to pelvic endometriosis, where consensus MRI protocols and structured reporting have been developed, thoracic localization still lacks validated standards of interpretation [46].

Current guideline and consensus documents provide a useful conceptual framework for recognizing endometriosis as a systemic disease with possible extrapelvic manifestations. However, recommendations specifically addressing thoracic endometriosis remain largely based on expert opinion, retrospective evidence, and extrapolation from pelvic endometriosis, rather than on validated thoracic-specific diagnostic pathways. Therefore, these documents should be interpreted as clinically useful frameworks rather than definitive evidence-based protocols.

This lack of standardization represents one of the most significant methodological limitations and, at the same time, a key area for future research.

### 8.4. Histopathological and Biological Uncertainty

Although histopathological confirmation is traditionally considered the gold standard of diagnosis, in thoracic endometriosis it is often limited by stromal-dominant or fibrotic lesions without the presence of classical glandular structures [41]. Inconsistent use of immunohistochemical markers (CD10, ER, PR) further contributes to variability in diagnostic sensitivity [41,42]. This biological heterogeneity explains why a negative histological finding does not exclude the disease and confirms the need for integrated clinical and surgical correlation.

### 8.5. Lack of Long-Term and Multidisciplinary Data

Most published studies have limited follow-up periods and small patient numbers, which makes assessment of long-term outcomes, fertility, and quality of life difficult [39,44]. A particularly notable gap exists between thoracic and gynecological series, despite the fact that TES represents a manifestation of a systemic disease [37,38,39]. Without integrated multidisciplinary registries and prospective cohorts, progress in this field remains incremental and center-specific.

### 8.6. Directions for Future Research

Priorities for future research include:

- development of a uniform phenotypic classification of TES;
- standardization of surgical protocols with clear criteria for diaphragmatic intervention;
- validation of structured MRI reporting for thoracic localization;
- prospective multicenter cohorts with long-term follow-up;
- systematic inclusion of fertility and quality-of-life parameters in outcome analyses [39,43,44,45,46].

Only within such a methodological framework will it be possible to move from experience-driven management toward an evidence-based model of clinical decision-making. Recent literature increasingly emphasizes the variability of clinical and radiological presentations of TES, as well as the limitations of standard diagnostic approaches, shifting the focus from isolated findings toward integrative interpretation within the clinical context. Existing guidelines provide a general framework for diagnosis and management, but they still leave considerable room for interpretation, particularly in atypical and subclinical forms of the disease [3,18,46].

### 8.7. Synthetic Conclusion of Chapter 8

The available evidence on thoracic endometriosis remains limited by phenotypic heterogeneity, variability in therapeutic approaches, and the absence of standardized protocols, underscoring the need for more rigorous prospective and multidisciplinary research.

## 9. Conclusions

TES represents a complex and probably underrecognized clinical entity whose diagnostic challenges arise from phenotypic heterogeneity, limited specificity of imaging findings, and a lack of standardized thoracic-specific diagnostic pathways. Its recognition depends on a high level of clinical suspicion and careful correlation of clinical history, temporal association of symptoms with the menstrual cycle, imaging findings, and, when appropriate, surgical and histopathological assessment.

Available evidence suggests that no single diagnostic modality is sufficient for reliable etiological confirmation in all cases. Imaging plays an important role in detecting and mapping thoracic manifestations, but its findings should be interpreted within the clinical and gynecological context. In selected patients, especially those with recurrent symptoms or suspected diaphragmatic involvement, intraoperative assessment may provide important diagnostic information, particularly when combined with systematic evaluation of the diaphragm and histopathological correlation.

Timely recognition of TES may have relevant therapeutic implications, including more appropriate planning of surgical intervention, consideration of hormonal suppression, and reduction in recurrence risk. However, therapeutic decisions should remain individualized, as the available evidence is still largely based on retrospective studies, small case series, and expert-based recommendations.

Further progress requires phenotype-stratified, multicenter, and multidisciplinary research with more standardized reporting of imaging findings, surgical observations, histopathological confirmation, treatment strategies, recurrence, fertility outcomes, and quality-of-life measures. Until more robust evidence and validated diagnostic pathways become available, an integrated clinical–radiological–surgical approach should be regarded as a pragmatic decision-support framework that may help improve recognition, reduce diagnostic delay, and guide multidisciplinary management of thoracic endometriosis.

## Figures and Tables

**Table 1:** Clinical phenotypes of thoracic endometriosis and key diagnostic implications.

Phenotype	Dominant Site	Key Clinical Clue	Main Diagnostic Pitfall	Suggested Diagnostic Focus
Catamenial pneumothorax	Pleura/diaphragm	Recurrent right-sided pneumothorax around menstruation	Misclassified as primary spontaneous pneumothorax	Ask explicitly about menstrual timing; evaluate diaphragm
Endometriosis-associated non-cyclic pneumothorax	Pleura ± diaphragm	Recurrent pneumothorax in a woman of reproductive age, even without clear cyclicity	TES excluded because symptoms are not strictly catamenial	Detailed gynecologic history; consider TES if recurrent/right-sided
Catamenial hemothorax	Pleura	Hemorrhagic pleural effusion during menstruation	Classified as idiopathic hemothorax after excluding trauma/coagulopathy	Assess cyclicity and possible pleural/diaphragmatic disease
Catamenial hemoptysis	Lung parenchyma/endobronchial lesions	Recurrent hemoptysis synchronized with menstruation	Treated as infection, vasculitis, or malignancy workup only	Correlate symptoms with cycle; CT/MRI during symptomatic phase
Diaphragmatic endometriosis	Diaphragm	Thoracic/shoulder/upper abdominal pain or recurrent right-sided events	Missed on CT/MRI and detected only intraoperatively	Careful right hemidiaphragm assessment; consider VATS when suspicion persists

Note: The presented phenotypes do not represent a formal classification, but rather a practical clinical framework based on dominant anatomical localization and presentation patterns. Individual manifestations may overlap, and the absence of strict cyclicity does not exclude thoracic endometriosis, particularly in women with recurrent right-sided pneumothorax [7,8,9,10,11]. This framework is intended to support clinical suspicion and diagnostic reasoning rather than to define rigid diagnostic categories.

**Table 2:** Imaging modalities in thoracic endometriosis: diagnostic role and principal limitations.

Modality	Main Diagnostic Role	Typical Findings	Main Limitation in TES
Chest radiography (CXR)	Initial evaluation of acute thoracic events	Pneumothorax, pleural effusion, hydropneumothorax	No etiological specificity; may be normal between episodes
Chest CT	Assessment of pleural and parenchymal abnormalities	Pneumothorax, hemothorax, ground-glass opacities, subpleural changes	Imaging findings are nonspecific and overlap with inflammatory, vascular, and neoplastic conditions
MRI (chest/diaphragm)	Detection of hemorrhagic and diaphragmatic lesions	T1-hyperintense lesions, diaphragmatic nodules or defects	Diagnostic yield depends on lesion timing and size; superficial or microscopic lesions may remain occult
VATS	Direct visualization and histological confirmation	Pleural implants, diaphragmatic fenestrations, endometriotic lesions	Invasive procedure; reserved for selected patients with persistent suspicion or recurrent disease

Note: Imaging modalities in TES differ primarily in their ability to detect complications, characterize tissue, and support etiological suspicion. None of the available imaging methods alone provides sufficient specificity for definitive diagnosis, which justifies the need for an integrated clinical–radiological–surgical approach [15,16,17,18,20,21,22]. Abbreviations: CT, computed tomography; CXR, chest radiography; MRI, magnetic resonance imaging; TES, thoracic endometriosis syndrome; VATS, video-assisted thoracoscopic surgery.

**Table 3:** Imaging versus thoracoscopic exploration in thoracic endometriosis.

Diagnostic Domain	Imaging (CXR/CT/MRI)	VATS
Detection of acute thoracic events	High	High
Etiological characterization	Limited	High
Detection of microscopic/superficial lesions	Low	Possible
Influence of menstrual-cycle timing	Significant	Less pronounced
Therapeutic capability	No	Yes

Note: While imaging modalities provide essential morphological assessment and support clinical suspicion, definitive etiological confirmation of thoracic endometriosis frequently relies on thoracoscopic visualization and histopathological correlation. Accordingly, imaging and VATS should be considered complementary rather than competing diagnostic approaches [17,18,20]. Abbreviations: CT, computed tomography; CXR, chest radiography; MRI, magnetic resonance imaging; VATS, video-assisted thoracoscopic surgery.

**Table 4:** Differential diagnostic considerations in women of reproductive age presenting with spontaneous pneumothorax.

Condition	Typical Clinical Context	Characteristic Imaging Pattern	Temporal Association	Feature Favoring TES
Primary spontaneous pneumothorax	Acute chest pain/dyspnea without known lung disease	Apical blebs, subpleural bullae	No cyclicity	Absence of menstrual association
Secondary pneumothorax (e.g., BHD, LAM)	Underlying cystic or systemic disease	Diffuse cystic lung abnormalities	Independent of menstrual cycle	Extrapulmonary/systemic manifestations
Catamenial pneumothorax	Recurrent episodes in women of reproductive age	Often nonspecific CT findings; possible diaphragmatic/subpleural abnormalities	Perimenstrual	Right-sided predominance with symptom cyclicity
Pulmonary thoracic endometriosis	Cyclic hemoptysis ± chest pain	Transient ground-glass opacities or infiltrates	Synchronous with menstruation	Reproducible temporal symptom pattern
Infectious disease (e.g., tuberculosis, abscess)	Fever and systemic inflammatory symptoms	Consolidation, cavitation	Progressive/non-cyclic	Laboratory and microbiological confirmation
Malignancy	Progressive clinical course	Persistent mass or pleural effusion	No cyclicity	Lack of interval regression

Note: Differential diagnosis of spontaneous pneumothorax in women of reproductive age should integrate temporal symptom pattern, laterality, imaging findings, and gynecological history. Temporal association with menstruation remains the strongest clinical clue suggesting thoracic endometriosis, whereas imaging findings alone rarely provide definitive etiological differentiation [16,18,20]. Abbreviations: BHD, Birt–Hogg–Dubé syndrome; LAM, lymphangioleiomyomatosis; TES, thoracic endometriosis syndrome; CT, computed tomography.

**Table 5:** Phenotype-oriented therapeutic strategies in thoracic endometriosis.

Therapeutic Strategy	Best Suited for	Therapeutic Rationale	Main Advantage	Main Limitation
Hormonal suppression	Mild disease, non-surgical candidates, or postoperative adjuvant therapy	Suppresses cyclic hormonal stimulation of ectopic tissue	Non-invasive; may reduce recurrence	Does not correct diaphragmatic or pleural defects
VATS with lesion resection	Recurrent pneumothorax/hemothorax or persistent clinical suspicion	Enables direct visualization and removal of visible lesions	Diagnostic and therapeutic in the same procedure	Recurrence may occur if diaphragmatic disease is missed
Diaphragmatic repair/resection ± mesh	Visible diaphragmatic defects, fenestrations, or recurrent right-sided events	Corrects structural diaphragmatic substrate	Associated with lower recurrence in available series	Technically demanding; evidence mainly retrospective
Pleurodesis	Recurrent pneumothorax, especially when no clear implant is identified	Promotes pleural symphysis and reduces pleural recurrence	Widely available and technically straightforward	Does not address hormonal or diaphragmatic drivers
Combined surgical and hormonal approach	Recurrent, complex, or diaphragm-associated TES	Combines structural correction with hormonal suppression	Most consistent long-term disease control	Requires multidisciplinary follow-up and individualized planning

Note: Therapeutic strategies in TES should be interpreted as phenotype-oriented management options rather than as a validated treatment algorithm. Current evidence is largely based on retrospective series, case reports, and expert consensus. Available data suggest that combined surgical management and postoperative hormonal suppression may provide the most durable recurrence control, particularly when diaphragmatic involvement is present [29,34,36,38,40]. Abbreviations: TES, thoracic endometriosis syndrome; VATS, video-assisted thoracic surgery.

## Data Availability

Data presented in this study are available from the corresponding author upon request.

## Abbreviations

The following abbreviations are used in this manuscript:

TES	Thoracic endometriosis syndrome
CP	Catamenial pneumothorax
CHt	Catamenial hemothorax
CHp	Catamenial hemoptysis
CS	Catamenial syndrome
CXR	Chest X-ray
CT	Computed tomography
MRI	Magnetic resonance imaging
VATS	Video-assisted thoracoscopic surgery
COC	Combined oral contraceptive
ER	Estrogen receptor
PR	Progesterone receptor
CD10	Cluster of differentiation 10
